# The Role of Cholesterol in Amyloidogenic Substrate Binding to the γ-Secretase Complex

**DOI:** 10.3390/biom11070935

**Published:** 2021-06-24

**Authors:** Urszula Orzeł, Jakub Jakowiecki, Krzysztof Młynarczyk, Sławomir Filipek

**Affiliations:** Faculty of Chemistry, Biological and Chemical Research Centre, University of Warsaw, 02-093 Warsaw, Poland; u.orzel@student.uw.edu.pl (U.O.); jjakowiecki@chem.uw.edu.pl (J.J.)

**Keywords:** Alzheimer’s disease, γ-secretase complex, membrane proteolysis, amyloid precursor protein, cholesterol

## Abstract

Alzheimer’s disease is the most common progressive neurodegenerative disorder and is characterized by the presence of amyloid β (Aβ) plaques in the brain. The γ-secretase complex, which produces Aβ, is an intramembrane-cleaving protease consisting of four membrane proteins. In this paper we investigated the amyloidogenic fragments of amyloid precursor protein (substrates Aβ_43_ and Aβ_45_, leading to less amyloidogenic Aβ_40_ and more amyloidogenic Aβ_42_, respectively) docked to the binding site of presenilin, the catalytic subunit of γ-secretase. In total, we performed 9 μs of all-atom molecular dynamics simulations of the whole γ-secretase complex with both substrates in low (10%) and high (50%) concentrations of cholesterol in the membrane. We found that, at the high cholesterol level, the Aβ_45_ helix was statistically more flexible in the binding site of presenilin than Aβ_43_. An increase in the cholesterol concentration was also correlated with a higher flexibility of the Aβ_45_ helix, which suggests incompatibility between Aβ_45_ and the binding site of presenilin potentiated by a high cholesterol level. However, at the C-terminal part of Aβ_45_, the active site of presenilin was more compact in the case of a high cholesterol level, which could promote processing of this substrate. We also performed detailed mapping of the cholesterol binding sites at low and high cholesterol concentrations, which were independent of the typical cholesterol binding motifs.

## 1. Introduction

Despite recent advances in research, the mechanism of Alzheimer’s disease (AD) is not yet fully understood. However, the extracellular presence of amyloid β (Aβ) plaques, together with soluble oligomeric forms of Aβ, are considered the major cause of AD [1,2]. The currently used therapeutics temporarily ameliorate cognitive decline but are unable to stop or reverse the progression of dementia. Several drug discovery campaigns have been launched, especially to block the γ-secretase protease, the protein that makes a final cut and produces Aβ, but they all failed in clinical trials [3,4]. Therefore, more studies are needed to investigate how substrates bind to the active site of γ-secretase and what factors influence that binding and subsequent processing. 

### 1.1. APP Processing

**Figure 1:**
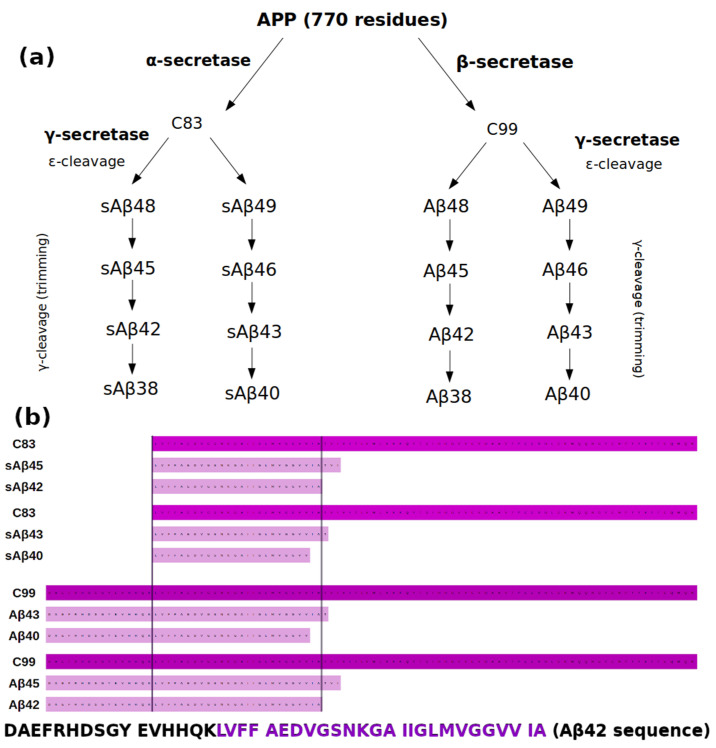
(**a**) The nonamyloidogenic (after α-secretase cleavage) and amyloidogenic (after β-secretase cleavage) pathways of amyloid precursor protein (APP); (**b**) the sequence multiple alignment of selected substrates and products of APP. At the bottom of the figure, the Aβ_42_ sequence is shown, including the sAβ_42_ sequence marked in violet.

The different isoforms of Aβ, the main constituent of the senile plaques, are produced by proteolytic cleavage from a larger precursor molecule called amyloid precursor protein (APP), a ubiquitous integral membrane protein that can undergo proteolytic processing by two distinct pathways: nonamyloidogenic and amyloidogenic. The γ-secretase substrates generally require shedding of their extracellular domains prior to intramembrane proteolysis. Shedding of APP by α- and β-secretases generates C83 (on the nonamyloidogenic pathway) or C99 (on the amyloidogenic pathway), respectively (Figure 1a), which are subject to cleavage by γ-secretase [5]. C83 forms a more stable complex with γ-secretase, and the products of its cleavage are soluble (hence the letter “s” before their names) and nontoxic. On the other hand, C99 forms a less stable complex with γ-secretase, and the products of its cleavage are toxic. They are longer by 16 residues compared to their counterparts from the nonamyloidogenic pathway (Figure 1b). 

C99 cleavage is mainly initiated at the alternative ε48 and ε49 sites, the latter being the major initial substrate cleavage site. After this cleavage, which releases the APP intracellular domain (AICD), proteolysis continues with the release of tri- and tetra-peptides after cleavage at alternate ζ- and γ-sites [2,6]. Successive cleavage along these pathways and some crossover between them leads to release of the Aβ peptides, including the most abundant Aβ_40_ and the minor Aβ_42_ [7,8]. This cleavage predominantly produces Aβ_40_ (the 40-amino-acid-long isoform) and Aβ_42_ (the 42-amino-acid-long isoform) at a ratio of 10:1. Aβ_42_ peptide is more hydrophobic and prone to forming aggregates, including oligomers, protofibrils, and finally amyloid fibrils, which are deposited as senile plaques in the brains of patients suffering from Alzheimer’s disease [9]. 

Although there is considerable conformational flexibility of the membrane part of monomeric C99 [10,11,12], including bending at its G_37_G_38_ hinge [13], only a few of these conformations eventually lead to cleavage. Growing evidence suggests that the efficiency and the specificity by which a substrate is recognized and cleaved may be governed by its overall structural dynamics in the binding site, possibly including local unfolding near the scissile bond. Molecular dynamics (MD) simulations could possibly reveal some aspects and correlations in the γ-secretase–Aβ complex. 

### 1.2. The γ-Secretase Structure

**Figure 2:**
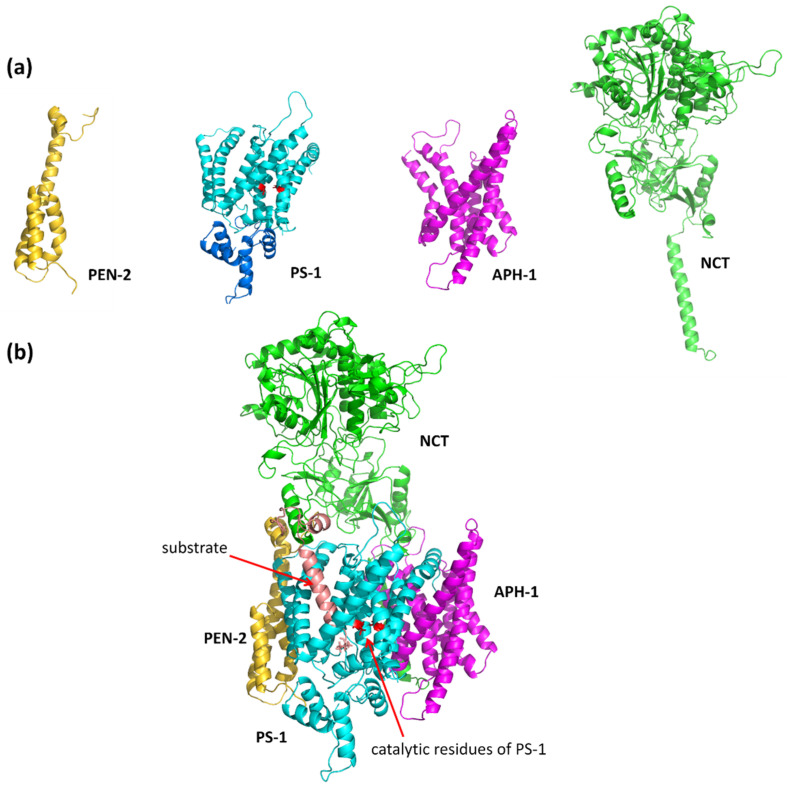
The structure of γ-secretase. (**a**) The individual components of the complex. PS-1 is shown with the modeled long cytosolic loop (residues 288–378, colored in blue) located between helices TM6 and TM7 that contains catalytic residues. The catalytic residues are shown in red. (**b**) The whole complex of γ-secretase together with docked substrate Aβ. Colors of proteins: PS-1 in cyan, APH-1 in purple, NCT in green, PEN-2 in yellow, and the Aβ substrate in salmon.

The γ-secretase complex consists of the catalytic subunit presenilin (PS-1), which is associated in a 1:1:1:1 stoichiometry with three subunits: PEN-2, APH-1, and NCT (Figure 2a). PEN-2 (presenilin enhancer 2) is composed of three membranous helices; however, two of them are half-helices, with a turn between them in the middle of the membrane. PS-1 contains nine transmembrane helices: TM1–TM6, forming the N-terminal part, and TM7–TM9, forming the C-terminal part. During maturation of the γ-secretase complex, the loop between helices TM6 and TM7 is cleaved, but both parts of PS-1 are located close together. APH-1 (anterior pharynx defective 1) is composed of seven transmembrane helices with relatively short loops between them, while NCT (nicastrin) has only one transmembrane helix and a very large ectodomain, which is thought to play a role in substrate recognition. The recently published cryo-EM structure (Protein Data Bank PDB id: 5FN2, resolution 4.2 Å) of the γ-secretase complex contains all of them (APH-1 in A form) and also a substrate (Figure 2b) [14,15]. Nearly all coordinates of TMs of γ-secretase have been assigned; however, the structure also revealed a disorder in its catalytic subunit presenilin (PS-1). Extensive structural dynamics of γ-secretase have also been demonstrated by transmission electron microscopy [16]. The newer cryo-EM structure of GS with a long APP-C83 substrate, i.e., before a first cut by γ-secretase, solved with a better resolution (PDB id: 6IYC, resolution 2.6 Å) [17], generally confirmed the previously determined structure (Figure S1). 

γ-secretase has been proposed to predominantly reside within cholesterol-rich lipid rafts [18,19,20]. The essential component of lipid rafts is cholesterol, and its high concentration in the brains of AD patients has been correlated with increased γ-secretase activity [21]. It was also observed that Aβ production was proportional to the abundance of cholesterol in various lipid membranes [22]. Thus, the membrane composition likely has an impact on the recognition and cleavage of substrates by γ-secretase [23]. 

### 1.3. Recent Studies Involving MD Simulations of the γ-Secretase Complex

Hitzenberger and Zacharias [24] performed molecular dynamics (MD) simulations to study the global dynamics and conformational transitions of γ-secretase without a substrate. They also studied distributions of water and POPC (1-palmitoyl-2-oleoyl-*sn*-glycero-3-phosphocholine) lipids, without cholesterol, in and around the transmembrane domains. Simulations were performed on the full enzyme complex (1μs MD simulation) and on the membrane-embedded parts alone (3.5 μs MD simulation). PDB structure 5FN2 of γ-secretase was used and the lacking loop between helices TM6 and TM7 was restored and cleaved to enable the simulation of the matured complex. They found that there are spots at the transmembrane surface of γ-secretase that facilitate the strong binding of lipids. The same authors [25] performed 12 μs MD simulations in free C99 and the cleaving intermediates Aβ_49_, Aβ_46_, and Aβ_43_ in complex with the enzyme to study the possible mechanisms responsible for the repositioning of the substrate at the binding site. They performed simulations in a POPC bilayer without cholesterol.

Aguayo-Ortiz and Dominguez [26] investigated the APH-1A component of γ-secretase using MD and umbrella sampling employing an all-atom model of whole γ-secretase without lacking loop TM6–TM7 based on cryo-EM structure 5FN2. They used a POPC membrane without cholesterol. The obtained results suggested that APH-1A allowed for the influx of extracellular cations into a central hydrophilic cavity but could not transport them into the intracellular space. In other research from the same group [27], they studied the influence of the membrane lipid composition on the structure and activity of γ-secretase. For that, they built a coarse-grain (CG) model of the protease based on the 5FN2 cryo-EM structure. Six model membranes of different thickness and properties, POPC, POPE, POPA, DLPC, DPPC, and DGPC in homogenous membranes, as well as three heterogenous membranes of POPC with various concentrations of cholesterol (20%, 40%, and 60%), were used. Additionally, a few 200 ns all-atom MD simulations were performed with homogenous and heterogenous (40% cholesterol) models of the membrane. By calculating the lipid residence times and interactions, they identified potential lipid and cholesterol binding sites. They also found that PS-1 adapts its conformational state in response to the hydrophobic mismatch with the lipid bilayer.

Recently, Bhattarai et al. [28] performed all-atom MD simulations using Gaussian accelerated molecular dynamics (GaMD). GaMD employs an enhanced sampling technique in which a harmonic potential is added to smooth the potential energy surface and thus reduce the energy barriers. They used the recently determined cryo-EM structures of γ-secretase with APP-C83 (PDB id: 6IYC) [17] and Notch fragment (PDB id: 6IDF) [29]. The POPC model membrane without cholesterol was used for all simulations with mutated and wild-type γ-secretase and with different substrates, including mutated APP. The simulations lasted from 300 ns to 2 μs. The investigators focused on the influence of APP mutations on cleavage site preferences.

### 1.4. Our Investigations

**Figure 3:**
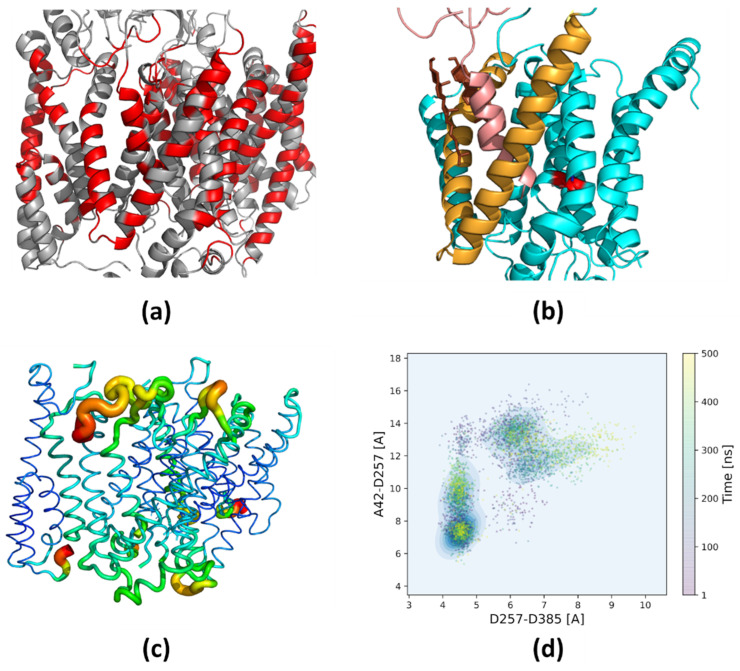
An illustration of the most important findings described in the paper. (**a**) Detailed map of the cholesterol binding sites; (**b**) repositioning of the substrate by its direct contact with cholesterol, preventing the bending of PS-1 helix; (**c**) differences in flexibility of the substrates in the binding site of PS-1, especially at a high level of cholesterol; (**d**) differences between substrates in terms of the compacting of the active site.

In order to observe the possible influence of the membrane composition on the stability and position of Aβ in the binding site of presenilin, we performed all-atom MD simulations with two different concentrations of cholesterol, 10% and 50%, in the membrane. We investigated the whole complex of γ-secretase in the all-atom water/membrane environment to obtain an unbiased picture. Considering the structurally similar amyloidogenic substrates, Aβ_43_ and Aβ_45_, leading in the next cut to less toxic Aβ_40_ and more toxic Aβ_42_ peptide, respectively, allowed us to see similarities and differences in their binding and movements and how cholesterol influences their behavior. By conducting a large number of long MD simulations, we created a detailed map of the cholesterol binding sites in all membrane components of γ-secretase (Figure 3a). It was found that at a high cholesterol level there is a repositioning of the substrate by its direct contact with two molecules of cholesterol, preventing the bending of helix TM3 of PS-1 (Figure 3b). By comparing the root-mean-square fluctuations of individual residues, we found statistically valid differences in the flexibility of Aβ_43_ and Aβ_45_ at the binding site of PS-1, especially at a high level of cholesterol (Figure 3c). Finally, we found differences between Aβ_43_ and Aβ_45_ in the active site of PS-1 in terms of the compacting of the active site and the distance between the scissile bond and the catalytic residues (Figure 3d). 

## 2. Materials and Methods

### 2.1. Missing Fragments’ Modeling

To construct a complete model of the structure of the human γ-secretase complex, we used a cryo-EM structure (PDB id: 5FN2) [15] and, first of all, the complex was converted to the wild type so the mutations introduced to improve protein stability were reversed. In 5FN2, not only the transmembrane helix TM2 is fully resolved but also a large part of the loop connecting TM6 to TM7 is visible. We modeled the missing large fragment of about 90 amino acids (residues 288–378) from a long intracellular loop (residues 262–381). The loop was autocatalytically cleaved to make a mature γ-secretase complex, so both lacking fragments, 288–298 and 299–378, were modeled independently. We used the BuildLoop function in YASARA Structure v.20.4 program [30] for modeling the missing fragments: 10 variants of the 288–298 fragment and 50 variants of the 299–378 fragment were constructed. The BuildLoop command searches the Protein Data Bank (PDB) for stretches of residues with start and end points that superpose well on the two atom selections and transfers the top scoring hits to copies of the selected object. The loops are also closed so that the covalent geometry around the anchor points is not negatively affected [31]. Loops are built based on a search through a nonredundant set of the PDB (90% sequence identity cutoff, resolution better than 2.5 A). The top scoring loops are chosen so that the root-mean-square deviation (RMSD) for superposing the anchor atoms is low, the sequence and secondary structure is similar to the Sequence (the loop sequence) and SecStr (set Any) parameters, and the sum of backbone bumps with other atoms in the same object is smaller than the Bumpsum parameter, which was set to a default value of 1 Å. Later, after MD equilibrations, the final structure of the modeled long cytoplasmic loop between helices TM6 and TM7 had a different and more compact structure compared to the initial structure from YASARA. 

The hydrogen atoms were added in YASARA, at a pH of 7.4 and with the optimization of the hydrogen bond network. The counterions Na^+^ and Cl^−^ were added to make the system neutral, and we set the salt concentration to a physiological level of 0.15 M. One of the catalytic residues, D385, was protonated to create the proper catalytic environment for this aspartic protease enzyme. The membrane with a proper cholesterol concentration was prepared in CHARMM-GUI [32]. The γ-secretase structures with the two best variants of the 288–378 region of the loop TM6–TM7 were verified by all-atom MD simulation and, after equilibrating for 700 ns, the more stable structure was selected for the substrate docking. The resulting loop contained two small helices that corresponded well with the small helix already existing in this loop in the experimentally determined structure. The Ramachandran plot indicated that the equilibrated structure of γ-secretase is stable and contains a large number of secondary structure elements (Figure S2a). Most of the extended parts belong to NCT, as was seen for the complex without NCT (Figure S2b), as this protein contains a large number of β-sheets in its large ectodomain. The Ramachandran plots for the initial cryo-EM structure (PDB id: 5FN2) (Figure S2c,d) are very similar to those for the equilibrated structure of γ-secretase. 

### 2.2. Substrate Docking

To prepare the APP substrate for docking, we used APP-C99 (the 99 amino acid C-terminal fragment of APP) structures modeled by Pantelopulos et al. [10]. They performed simulations of monomeric wild-type C99 embedded in a membrane modeled with the GBSW implicit solvation method [33]. They used replica-exchange molecular dynamics (REMD) [34] with 16 replicas and performed C99 simulations in 30, 35, and 40 Å thick membranes. C99 ensembles included structures with metastable α-helices and β-strands in their N-termini. α-helical domains are thought to be nicastrin association sites, while β-strand structures could probably seed amyloid oligomerization on the membrane surface. To refine structures in the complex with γ-secretase, we chose a few with different N-termini. We also removed the C-terminal residues in order to have Aβ_43_ or Aβ_45_ structures. Then, every β-amyloid peptide (Aβ) was slightly unfolded at the C-terminus using interactive MD in YASARA Structure [30] using a NOVA force field [35], and such simulations were performed in vacuum, keeping the rest of Aβ frozen.

A docking procedure was done in the ICM-Pro v.3.8 program [36]. The procedure is based on the Fast Fourier Transform (FFT) docking method and contains two stages. The first stage uses a simplified scoring function representing steric fit and hydrophobic/hydrophilic contact matching. FFT is then used for a translational search, using a systematic search of rotations from 60 × 27 (coarse) to 256 × 125 (fine) orientations. The second stage rescores the top 3000–20,000 solutions with a more accurate energy function, including electrostatics and SAS-based (solvent-accessible surface) solvation. The conformations are then clustered using contact fingerprints.

In ICM-Pro, the interaction energy of the complex is calculated by grid potentials, using an ICM-DISCO docking procedure with fully flexible ligand side-chains [37]. The following energy terms are precalculated on a grid surrounding the 10 Å vicinity of the whole receptor (in our case, PS-1) and included in the interaction energy: the truncated van der Waals potential (maximum value 1.0 kcal/mol), the electrostatic potential corrected for the solvation effect, the hydrogen-bonding potential, and the hydrophobicity potential. The final scoring function also includes the solvation energy based on atomic solvent-accessible surfaces. 

The substrates Aβ_43_ and Aβ_45_ were docked to the structure of PS-1, the catalytic subunit of γ-secretase, which was taken from the equilibrated γ-secretase complex (as described in Section 2.1). The receptor focus for substrate docking was set only at the catalytic residues D257 and D385. The number of poses selected in each docking was 5000. Exemplary docking, showing the most populated clusters and the poses with the best scores, is shown in Figure S3. The structures in the most populated clusters also had the best scores. After initial pose clustering, the representative structures from the five most populated clusters underwent a structural refinement with ligand-flexible side chains (Figure S4). To determine the maximum diversity of substrate poses, the maximally distinct poses within the lowest score poses, with their N-termini outside the membrane and not interfering with the other subunits of γ-secretase, were chosen for MD simulations (Figure S5).

### 2.3. Molecular Dynamics (MD) Simulations

The MD simulations of the γ-secretase complex with docked substrates were performed in the POPC bilayer (10% cholesterol) and also in the raft-like bilayer (50% cholesterol) to study the influence of the lipid composition of the membrane on Aβ conformational dynamics and binding to γ-secretase. We performed all-atom simulations using the AMBER 18 program [38] with standard all-atom force field CHARMM36 [39]. The TIP3P water model was employed, which was parametrized to use with CHARMM force fields. The whole modeled system contained about 260,000 atoms, including 200–250 lipid molecules (cholesterol and POPC): the raft-like membranes contained closer to 250 lipids, while regular membranes were closer to 200 lipids. The average dimensions of the periodic cell were 130 Å × 130 Å × 170 Å. The whole system in the membrane was subjected to a restrained energy minimization (5000 cycles). The first 2500 minimization cycles were performed with a steepest-descent method, and after that, the conjugate gradient was employed in the AMBER program. Next, a six-step equilibration was performed (375 ps total) at constant pressure and temperature (NPT ensemble; 310 K, 1 bar). During the equilibration, the restraints were released gradually until the last step (which lasted 100 ps), in which no restraints were used. In the production simulations, as well as in the last three equilibration steps, all bond lengths to hydrogen atoms were constrained using the SHAKE [40] algorithm, allowing us to use a longer time step of 2 fs instead of 1 fs. Van der Waals and short-range electrostatic interactions with a cutoff of 12 Å and a 10–12 Å F-switch function were used. Long-range electrostatic interactions were computed using the Particle Mesh Ewald [41] summation scheme. 

Simulations were conducted in a typical phospholipid bilayer composed of POPC and 10% (n/n) cholesterol, which was used in our previous studies. For simulation of the system in the lipid rafts, 50% (n/n) cholesterol was used. All simulations were performed in a timescale of 500 ns for Aβ_43_ and Aβ_45_, employing eight different conformations (described in Section 2.2) in two different concentrations of cholesterol. Additionally, two 500 ns MD simulations were performed for the γ-secretase structure with an APP-C83 substrate (PDB id:6IYC). The RMSD and radius of gyration plots for exemplary MD simulations of the substrate in 10% and 50% cholesterol are presented in Figure S6. The plots indicate the stability of the γ-secretase complex and that the movement of the ectodomain of NCT is independent of cholesterol concentration since the plots for the transmembrane parts of the complex are very similar. 

### 2.4. Programs for Making Figures and Analyses 

Pictures were prepared in PyMOL 1.8.4.0 open-source. The following Python v.3.6.4 libraries were used: MDAnalysis [42], Matplotlib, SciPy, and NumPy. Analysis of hydrogen bonds was done in MDAnalysis. The Matplotlib Pyplot library was used to visualize the data on charts. Calculations of distances were done with MDAnalysis. Salt bridge analysis was done using VMD [43] plugin saltbr. This tool considers a salt bridge to be formed if the distance between any of the oxygen atoms of acidic residues and the nitrogen atoms of basic residues are within the cutoff distance of 3.2 Å. The helix curvature was measured and visualized using the Bendix [44] tool in the VMD program. 

## 3. Results and Discussion

A series of 16 MD simulations was performed using different conformations and different poses of Aβ substrates (Figure S5), docked to the refined cryo-EM structure of γ-secretase (PDB id:5FN2) in 10% and 50% cholesterol POPC membranes. We defined the receptor focus for substrate docking at the catalytic residues of D257 and D385 only. No other residues were selected; however, all the best scored poses, and also poses from the most populated clusters, were found between helices TM2 and TM3 of PS-1 and not on the other side of PS-1. This indicates that this site is really employed for the final cuts of amyloid substrates. The average value of the final docking score and the binding energy was lower for Aβ_43_ compared with Aβ_45_, suggesting a better binding of Aβ_43_. Two additional simulations of the PDB id:6IYC γ-secretase structure containing the APP-C83 substrate, also using two different concentrations of cholesterol, were done. All simulations were completed in a timescale of 500 ns to investigate the amyloidogenic pathway by simulating the γ-secretase complex with Aβ_43_ (leading to less amyloidogenic Aβ_40_) as well as with Aβ_45_ (leading to more amyloidogenic Aβ_42_). 

### 3.1. Mapping of the Cholesterol Binding Regions

**Figure 4:**
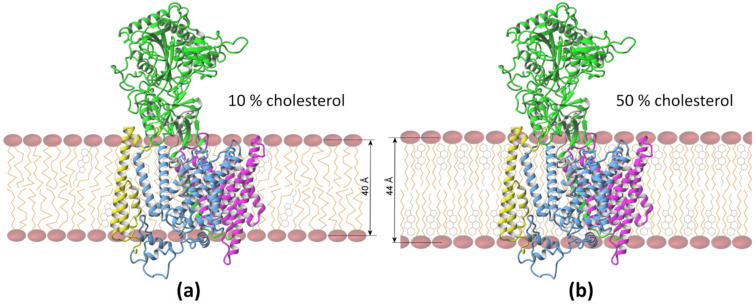
Position of the γ-secretase complex in the membrane and its average thickness taken from MD simulations. The same structure of the complex is shown in both panels for better visualization of its position. (**a**) The regular POPC membrane with 10% cholesterol and disordered lipid chains; (**b**) the raft-like bilayer with 50% cholesterol and nearly parallel lipid chains is on average 4 Å thicker.

**Figure 5:**
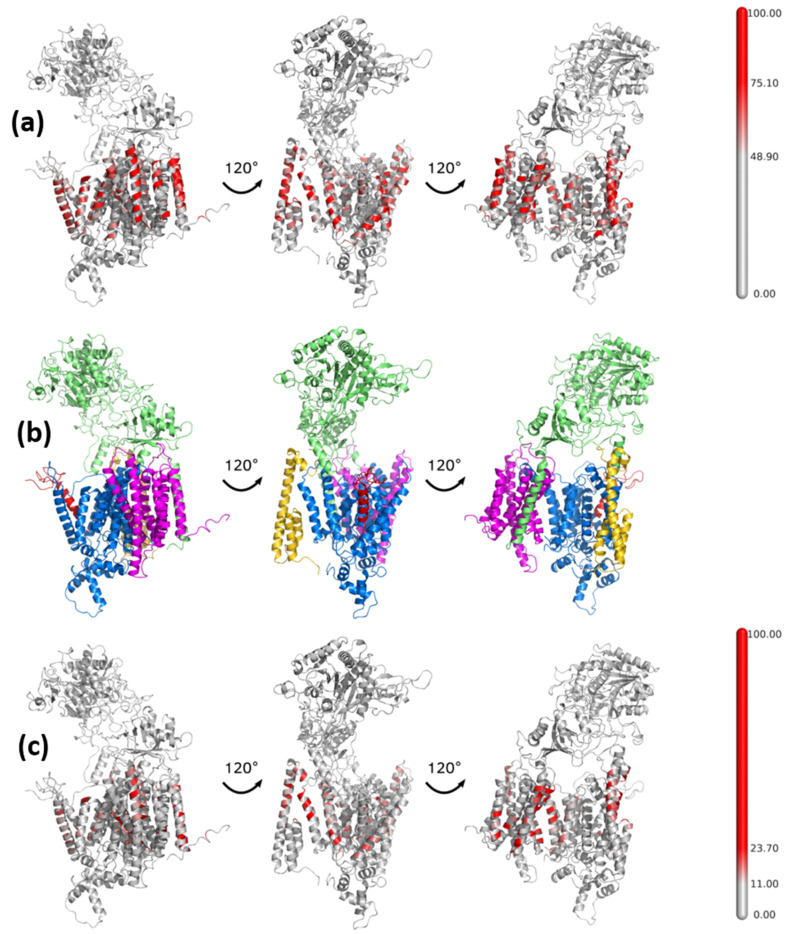
Location of areas of γ-secretase complex interacting with cholesterol molecules—the aggregated data from all simulations. Rotations of structure about 120° in each row; (**a**) 50% cholesterol, (**b**) the reference structure with the same rotations showing each molecule in separate color: PS-1 in blue, APH-1 in purple, nicastrin in green, PEN-2 in yellow, substrate in red; (**c**) 10% cholesterol. The bars on the left show the scale: the percentage of time during MD simulations of that particular residue being in contact with cholesterol.

The presence of a large number of cholesterol molecules in the membrane increases the width of the membrane, from 40 Å to 44 Å on average, mainly due to the elongation of long and hydrophobic lipid chains in contact with cholesterol (Figure 4), and influences the protein structure and dynamics. The contacts of cholesterol with the γ-secretase complex, calculated from all 16 MD simulations with amyloid substrates, are presented in Figure 5. There are more red areas, indicating close contacts with cholesterol in the 50% than in the 10% concentration, even though the scale indicating interactions with cholesterol is set to equalize both cases, i.e., the cutoffs for both concentrations are set to the 75th and 95th percentile for a given concentration. This suggests that the distribution of those values is very different in each concentration, i.e., the value for the 95th percentile is 115% and 53% larger than the value for 75th percentile for 10% and 50% cholesterol, respectively. In all conducted MD simulations at a high cholesterol level, we found that all proteins in the γ-secretase complex broadly interact with cholesterol (Figure 5a). The cholesterol present in both the upper and the lower part of the bilayer participates in interactions with the TM helices of all proteins of the protease, but the upper (extracellular) part is slightly more engaged in those interactions.

The substrate binding area, which can be identified by comparing it to the reference structure of the γ-secretase complex after the same rotations (Figure 5b), illustrates that the substrate is not in contact with cholesterol in its 10% concentration (Figure 5c), but this contact is achieved at a high cholesterol level (Figure 5a). The same is also true for the TM2 and TM3 transmembrane helices of PS-1, which flank the helix of the substrate. However, the adjacent helix TM4 of PS-1, which is separate from the rest of PS-1 and binds to PEN-2, is in persistent contact with cholesterol. This indicates that entering the cholesterol into the substrate binding site may be obstructed by amino acids from flanking helices TM2 and TM3 of PS-1. We have refrained from grouping the contact areas with cholesterol since there are so many tightly bound contacts. The distribution of residues of γ-secretase with the highest number of contacts with cholesterol is in qualitative agreement with that obtained by Aguayo-Ortiz et al. [27].

**Figure 6:**
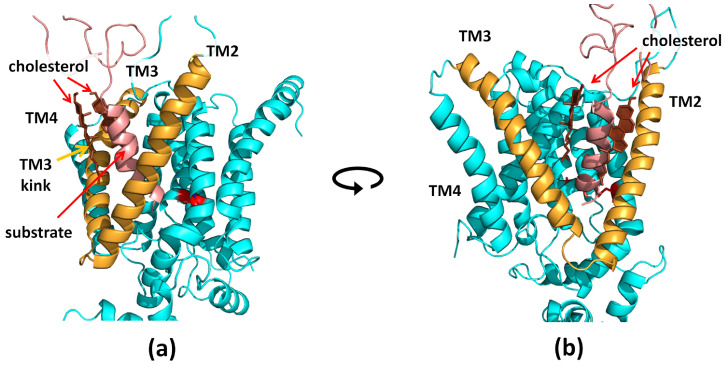
The presence of two cholesterol molecules (in brown) in the substrate binding site of PS-1 (in cyan) with extensive contacts with the substrate helix (in salmon) in the membrane with a high concentration of cholesterol. Helices TM2 and TM3 of PS-1, forming a triangle, are colored gold. The kink of TM3 is most visible in the left panel. The catalytic residues are shown in red. (**a**) Side view of the PS-1–substrate complex; (**b**) front view of the PS-1–substrate complex.

In our simulations, the presence of larger amounts of cholesterol directly influenced the position of the Aβ_43_ and Aβ_45_ substrates—typically at a higher cholesterol concentration, there were two cholesterol molecules interacting with Aβ (Figure 6), while with a low concentration of cholesterol, there was only one or no cholesterol molecule in contact with the substrate. The presence of cholesterol in such specific places between a helix of the substrate and helices TM2 and TM3 of PS-1 directly influenced the position of the substrate and possibly forced more amyloidogenic cleavage. 

**Figure 7:**
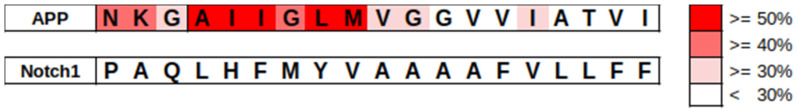
The sequence alignment of amyloid substrates (indicated as APP) in contact with cholesterol with the corresponding Notch1 sequence. The particular residues of substrates in contact with cholesterol are marked on the APP sequence in shades of red. The percentage values are averaged over all MD simulations with a 50% cholesterol concentration, and the color scale is shown on the right.

We also compared the fragment of the sequence of APP, containing residues in contact with cholesterol in all MD simulations with 50% cholesterol, with the sequence of Notch1, the other important substrate of γ-secretase (Figure 7). All residues in contact with the hydrophobic part of cholesterol (fragment AIIGLM) are hydrophobic but not aromatic. However, the equivalent fragment of Notch1 contains three aromatic residues. Additionally, the residues in the NK sequence of APP, which interact with the hydrophilic part of cholesterol, are not similar to the hydrophobic PA residues of Notch1. Such differences probably indicate a different pattern of interactions of cholesterol with Notch1 than was seen in our simulations of the Aβ substrates.

**Figure 8:**
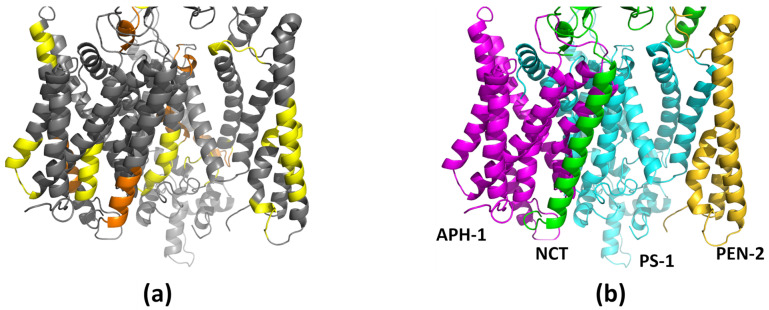
Location of the cholesterol binding motifs in γ-secretase. Only the membranous part of the complex is shown. (**a**) The cholesterol binding motifs CRAC (orange) and CARC (yellow); (**b**) the same orientation of γ-secretase with colored subunits is shown as a reference structure. The scheme of colors is the same as in Figure 2.

There are two well-known cholesterol binding motifs: CRAC (Cholesterol Recognition/interaction Amino acid Consensus, (L/V)-X_1–5_-Y-X_1–5_-(K/R)) and its almost exact reverse, CARC ((K/R)-X_1–5_-(Y/F)-X_1–5_-(L/V)). The γ-secretase complex contains many such motifs; however, only a few of them fulfill all requirements to become CRAC/CARC cholesterol binding domains, i.e., to be positioned within a transmembrane helix in a way that facilitates interactions with cholesterol from one of the leaflets. There are two putative CRAC/CARC domains in PS-1, three in APH-1, two in PEN-2, and one in the NCT TM domain (Figure 8). However, we found that those domains do not form the strongest cholesterol binding areas of γ-secretase TMs.

**Figure 9:**
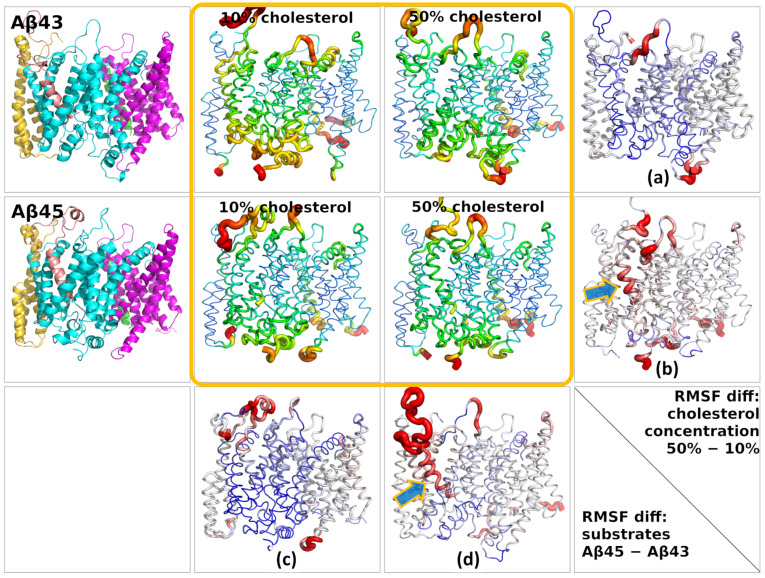
Root-mean-square fluctuations (RMSF) of particular residues of γ-secretase mapped on its 3D structure. The circled area of four panels contains the RMSF values coded by the color and size of backbone tube. Each central panel is an average of four simulations. The small RMSF values are in blue and higher ones are in green, yellow, and red. Only the membrane part of the complex is shown since the high RMSF values of flexible loops would mask much smaller fluctuations of the membrane part. (**a**) Differences in fluctuations between 50% and 10% cholesterol for the γ-secretase complex with Aβ_43_; (**b**) the same for Aβ_45_; (**c**) differences between fluctuations of the γ-secretase complex with Aβ_45_ and Aβ_43_ in membrane with 10% cholesterol; (**d**) the same for 50% cholesterol. Blue and orange arrows indicate the increased flexibility of the Aβ_45_ helix. Colors for RMSF differences are red for positive and blue for negative. For comparison purposes, the γ-secretase complexes are depicted in the left panels. The scheme of colors is the same as in Figure 2.

To compare the influence of cholesterol and type of substrate on the stability of the complex, we calculated the root-mean-square fluctuations (RMSF) for the γ-secretase–Aβ complex (Figure 9). The fragments protruding from the membrane, usually flexible loops, had the highest flexibility (the highest RMSF values), so we excluded them from the calculations. To get rid of global movements of subunits in relation to each other, we superimposed each subunit individually before calculating the RMSF values. In the case of the substrate, because of the high flexibility of its N-terminus, the superimposition was done based on the PS-1 structure. Comparing the fluctuations of Aβ_43_ in 10% and 50% cholesterol concentrations, we saw that the fluctuations of substrate and PS-1 were at the same level (Figure 9a); however, for Aβ_45_, the substrate fluctuations were larger than for PS-1 and involved two turns of the substrate helix (Figure 9b). Comparing Aβ_43_ and Aβ_45_ in 10% cholesterol (Figure 9c), we saw no difference in flexibility; however, in 50% cholesterol the flexibility of Aβ_45_ was much greater than that of Aβ_43_ (Figure 9d). Higher flexibility of Aβ_45_ was also seen for three turns of the substrate helix, not only its N-terminus. These results were based on four simulations for each case, so they are statistically sound. 

The increased flexibility of the Aβ_45_ substrate in 50% cholesterol compared with Aβ_43_ was probably caused by the mismatch of residues of substrate and the binding site of PS-1. On the other hand, the increased flexibility of Aβ_45_ substrate in 50% cholesterol compared with 10% cholesterol may have resulted from incompatibility between the substrate in the binding site and cholesterol molecules, which were not able to efficiently stabilize Aβ_45_. The substrates Aβ_43_ and Aβ_45_ were differently bound to PS-1 due to rotations of the substrate in the binding site, so different residues were found to be in contact with lipids and cholesterol.

The RMSF values for particular subunits of γ-secretase and the substrate, which were used to create Figure 9, are presented in the Supplementary Materials: (1) for the γ-secretase complex structure in the 10% cholesterol membrane (Figure S7); (2) the same in the 50% cholesterol membrane (Figure S8); (3) for the Aβ_43_ substrate in membranes with 10% and 50% concentrations of cholesterol (Figure S9); (4) the same for the Aβ_45_ substrate (Figure S10). The RMSF values are averaged over four MD simulations in each case. The most visible difference in terms of the fluctuations of residues between both substrates was for 50% cholesterol (Figure S8), where not only the N-terminus but also the whole helix of Aβ_45_ had higher RMSF values than the residues of the Aβ_43_ substrate. 

### 3.2. Positioning of Substrate in the Binding Site

**Figure 10:**
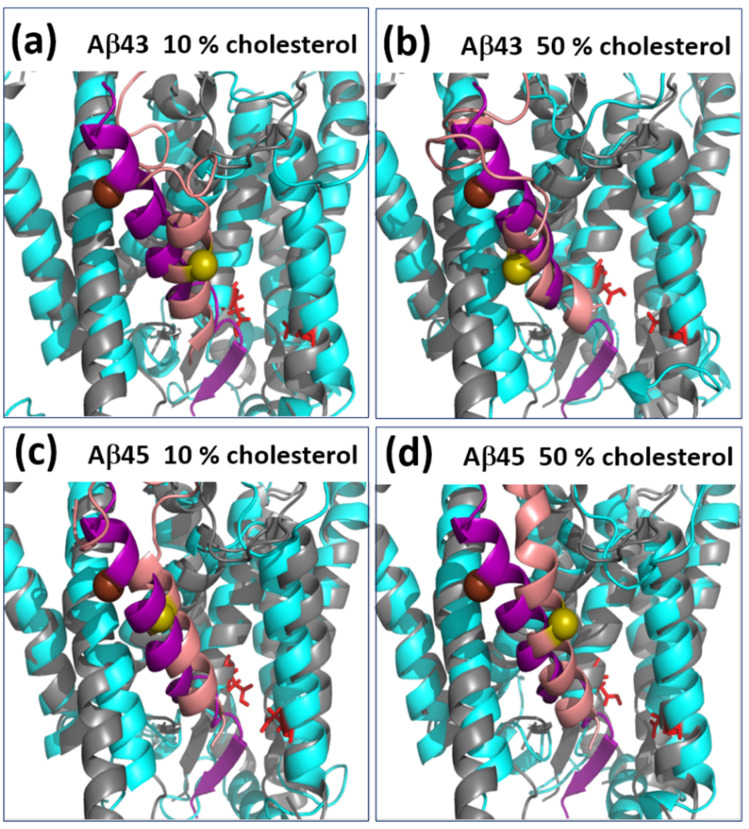
Comparison of cryo-EM structure (PDB id: 6IYC) with bound APP-C83 (purple helix and β-thread) with structures of Aβ peptides from exemplary MD simulations (helix in salmon). Olive and brown balls indicate the positions of the same residue to show possible rotations and movements of Aβ_43_/Aβ_45_ (olive) compared with APP-C83 (brown). (**a**) Superimposition of PS-1 with APP-C83 substrate and PS-1 with docked Aβ_43_ substrate after MD simulation of the γ-secretase complex in 10% cholesterol membrane; (**b**) the same in 50% cholesterol membrane; (**c**) superimposition of PS-1 with APP-C83 substrate and PS-1 with docked Aβ_45_ substrate after MD simulation of the γ-secretase complex in 10% cholesterol membrane; (**d**) the same in 50% cholesterol membrane.

The Aβ substrates were docked to the apo γ-secretase structure without any modification of the substrate binding site, and they easily reached the catalytic residue area with its C-terminus. We compared their docking poses with a position of APP-C83 in the recently determined structure of γ-secretase (PDB id: 6IYC) [17]. In 6IYC, the visible part of the substrate was a fragment of APP-C83 (APP residues 688–693 and 699–726), and its C-terminus was equivalent to the Aβ_55_ residue. After a comparison with our docking poses, we noted that the Aβ_43_/Aβ_45_ substrate position, taken from the final structures in MD simulations, was shifted and rotated (Figure 10). This is feasible since Aβ_43_/Aβ_45_ with a shorter length than C83 can be docked deeper into the active site. In the 6IYC structure, the substrate residue closest to the catalytic site is L49 (L720 in full-length APP), which is a fragment that undergoes epsilon cleavage. Aβ_43_ (Figure 10a,b) was bound approximately one helix turn deeper toward the active site than Aβ_45_ (Figure 10c,d). Cholesterol had no influence on the depth of the substrate pose in the binding site. We also performed 500 ns MD simulations of the γ-secretase complex of the 6IYC structure in the membranes with low and high cholesterol concentrations and found that APP-C83 was stable in both cases, including a small β-sheet formed by this substrate at its C-terminus and fragments of the loop between helices TM6 and TM7 of PS-1. 

### 3.3. Bending of TM3 PS-1 at Low Cholesterol Level

**Figure 11:**
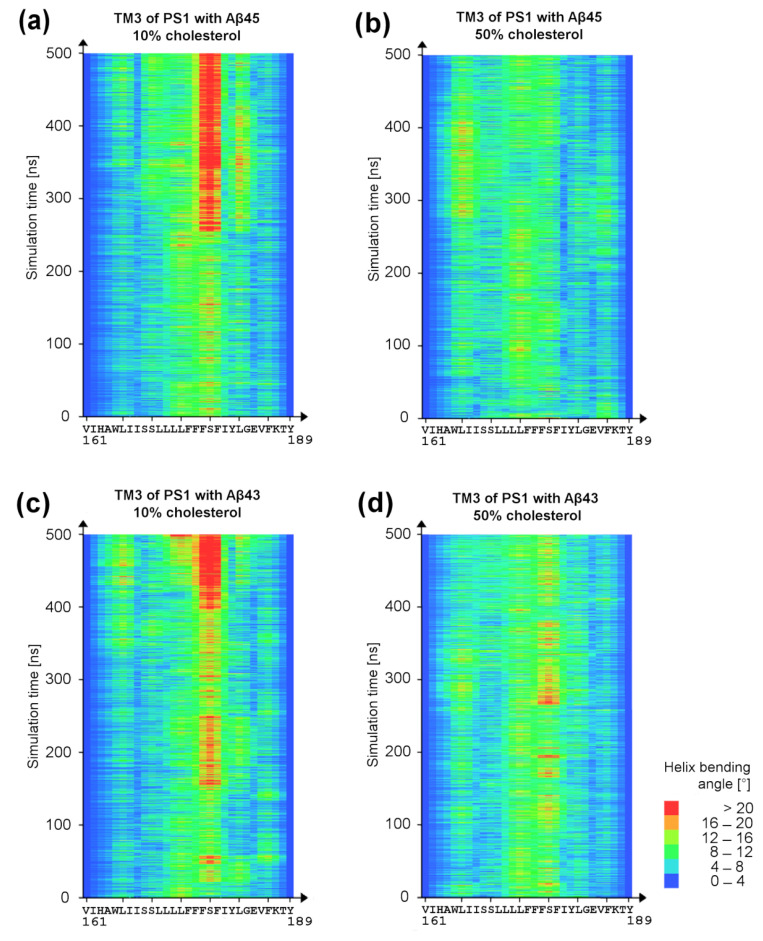
Bending of TM3 of PS-1 in low and high cholesterol concentrations in exemplary simulations. Helices TM2 and TM3 are in close contact with the substrate helix, and their positions can directly influence the position of the substrate at the binding site. (**a**) Bending angles at residues of TM3 PS-1 with Aβ_45_ substrate in 10% cholesterol membrane; (**b**) the same in 50% cholesterol membrane; (**c**) bending angles at residues of TM3 PS-1 with Aβ_43_ substrate in 10% cholesterol membrane; (**d**) the same in 50% cholesterol membrane.

The cholesterol influences the shape of PS-1 and, in particular, the bending of TM3 of PS-1 (Figure 6 and Figure 11). In 50% cholesterol, the kink is about half the size (about 12° difference at the end of those MD simulations presented in Figure 11). For the other two pairs of MD simulations with Aβ_43_ substrate, we did not observe statistically valid differences between TM3 bending angles, and the kink was small. Interestingly, in one pair of simulations with the Aβ_43_ substrate, we observed the opposite effect to that presented in Figure 11, i.e., a larger TM3 kink in higher cholesterol. However, after closer inspection of the interactions of cholesterol with the substrate, we found that, despite the large number of cholesterol molecules in the investigated system, there was only a small point of contact between the substrate Aβ_43_ and the tail of one cholesterol molecule, while, on the contrary, in 10% cholesterol there was a much stronger contact with the rings of the cholesterol molecule. Therefore, the presence of cholesterol (at least one molecule) with extensive interactions with the substrate helix is enough to prevent the bending of TM3. The straighter TM3 helix is presumably more stable and more precisely positions the substrate with its C-terminus toward the active site for making cleavage. We have not seen statistically valid differences between Aβ_43_ and Aβ_45_ substrates in the same concentration of cholesterol.

### 3.4. The Secondary Structure of the Substrate and Interactions with the Protease

**Figure 12:**
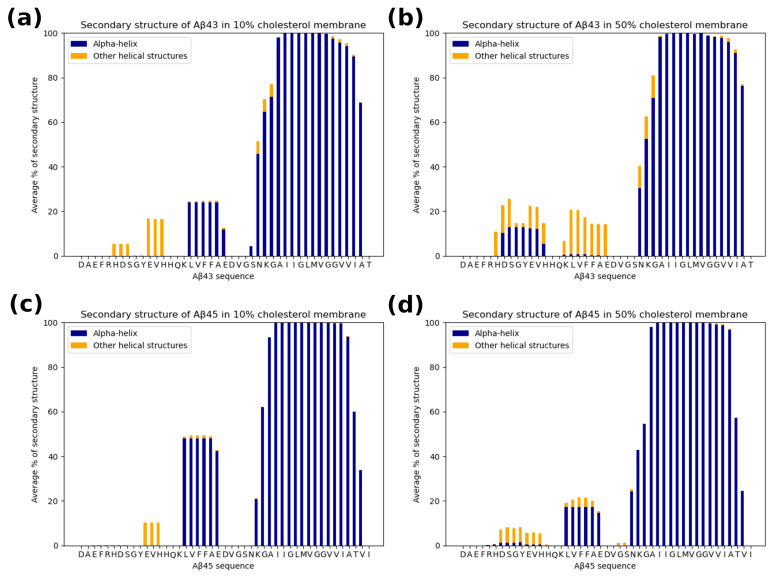
The average secondary structure of the substrate Aβ_43_ and Aβ_45_ during MD simulations in 10% and 50% of cholesterol. Each panel is an average of four MD simulations. (**a**) Aβ_43_ substrate in 10% cholesterol membrane; (**b**) the same in 50% cholesterol membrane; (**c**) Aβ_45_ substrate in 10% cholesterol membrane; (**d**) the same in 50% cholesterol membrane.

We also compared the secondary structures of the substrate at low and high cholesterol concentrations. The average secondary structure for each case (substrate/cholesterol) is shown in Figure 12. Only one residue was unfolded at the C-terminus for the Aβ_43_ case and two residues for Aβ_45_. Additionally, at a higher cholesterol level there is a smaller amount of regular α-helix for residues LVFFAE in the N-terminal part of the substrate. Due to the hydrophobic character of this fragment, it has contact with the membrane, enabling cholesterol to influence its structure. Both substrates, Aβ_43_ and Aβ_45_, have a similar secondary structure, and the effect of the increased cholesterol level on the diminishing helicity of the LVFFAE fragment is also similar. 

The N-terminus of the substrate is primarily disordered and can interact with PS-1 loops as well as with NCT. The residues of the substrate N-terminus form hydrogen bonds and also salt bridges. The number of hydrogen bonds formed between the substrate helix and γ-secretase ranges from 0 to 6 during the simulations (Figures S11 and S12). It increases to 9 when a full substrate (with N-terminus of Aβ_43_/Aβ_45_) is considered. Therefore, the hydrogen bonds seem to play a meaningful role in stabilizing the substrate in the binding site of PS-1 of the γ-secretase complex. However, it is not possible to distinguish differences in interactions between both substrates based on the number of hydrogen bonds created with PS-1 since they have a wide range of variability and indicate the large flexibility of substrates in the binding site of PS-1. 

### 3.5. Tracing Structural Changes in the Active Site

**Figure 13:**
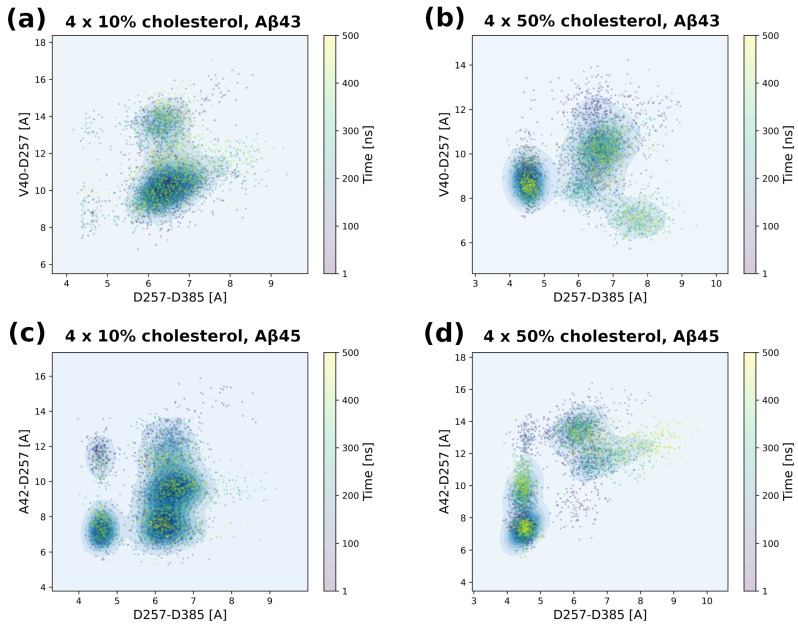
2D scatter plots showing distances between catalytic residues (horizontal axes) and distances between one of the catalytic residue (D257) and the peptide bond that is to be cleaved (vertical axes). Each panel is the average of four MD simulations. All simulations for each type of the system (Aβ_43_/Aβ_45_ and high/low cholesterol) were added to the grouping frames at the same time of simulation. All points are colored by the time of MD simulation from purple (0 ns) to yellow (500 ns). (**a**) MD simulations of γ-secretase complex with Aβ_43_ substrate in 10% cholesterol membrane; (**b**) the same in 50% cholesterol membrane; (**c**) MD simulations of γ-secretase complex with Aβ_45_ substrate in 10% cholesterol membrane; (**d**) the same in 50% cholesterol membrane.

To investigate the relative position of the scissile bond of the substrate to the catalytic residues of PS-1, we measured the distance from the carbonyl oxygen of that bond (V40 for Aβ_43_ or A42 for Aβ_45_) to one of the catalytic residues (D257). That distance was combined with the distance between the catalytic residues themselves (Figure 13). It is clear that a high cholesterol level brings the catalytic residues together—more yellow points in the area around 4.5 Å for the D257–D385 distance compared with the case of 10% cholesterol for both substrates; however, for Aβ_45_ the change from a distance of around 6.5 Å to 4.5 Å was more complete. There was also a change of distance between the catalytic residue and the substrate cleavage point. It was about 10 Å for Aβ_43_ in 10% cholesterol and diminished to 9 Å for 50% cholesterol. In the case of Aβ_45_, there were two equally populated areas of points around 10 Å and 8 Å for this distance in 10% cholesterol. However, in 50% cholesterol, these two areas disappeared, and there was a large concentration of points at around 7 Å for the distance of the scissile bond to the catalytic residue. A smaller distance means a greater probability of cleaving this bond; therefore, the high cholesterol level could promote the processing of Aβ_45_ over Aβ_43_, which is in agreement with experimental data on the levels of cholesterol in the brains of AD patients [21].

## 4. Conclusions

We performed all-atom MD simulations of the whole γ-secretase complex with substrates Aβ_43_ and Aβ_45_, which lead to less amyloidogenic Aβ_40_ and more amyloidogenic Aβ_42_ peptides in the next cut, in membranes with 10% and 50% concentrations of cholesterol. Apart from generating a detailed map of the potential cholesterol binding sites at low and high cholesterol levels, the simulations revealed differences in the behavior of structurally similar amyloidogenic substrates, Aβ_43_ and Aβ_45_, at the binding site of PS-1. At a high level of cholesterol, the active site was more compact in the case of Aβ_45_, while the average distance of the scissile bond to one of the catalytic residues was much shorter for Aβ_45_ than for Aβ_43_, indicating a higher probability of cleavage. Cholesterol was also important for positioning the substrate helix between helices TM2 and TM3 of PS-1, suggesting that high cholesterol can prevent the bending of TM3 for both substrates. Comparing the flexibilities of the γ-secretase complex with both substrates, we found that a part of the Aβ_45_ helix, having contact with lipids and cholesterol, is more flexible at a high cholesterol concentration. This could suggest a mismatch between residues of that part of Aβ_45_ and cholesterol molecules. Additionally, the flexibility of Aβ_45_ is statistically larger than that of Aβ_43_ at a high cholesterol level and could contribute to the faster processing of Aβ_45_ since a high level of cholesterol is a risk factor of AD. We hope that our investigations will shed light on the mechanisms of γ-secretase proteolysis and contribute to the development of effective drugs against AD. The generated cholesterol binding map of γ-secretase may be useful for studying the influence of the membrane composition on the mechanisms of the substrate cleavage.

## Data Availability

The data supporting reported results are deposited at Faculty of Chemistry, University of Warsaw, and are available upon request.

## Supplementary Materials

The following are available online at https://www.mdpi.com/article/10.3390/biom11070935/s1, Figure S1: Comparison of two structures of PS-1. (a) The cryo-EM structure (Protein Data Bank PDB id: 5FN2) with reconstructed long cytosolic loop (residues 288–378, colored in blue) between helices TM6 and TM7 containing catalytic residues. (b) The cryo-EM structure (PDB id: 6IYC) with lacking residues 292–375 of cytosolic loop between helices TM6 and TM7. The catalytic residues are colored in red, Figure S2: The Ramachandran plots for the structure of the γ-secretase complex without a substrate. (a) The structure after equilibration using 700 ns all-atom molecular dynamics (MD) simulation; (b) the same structure without NCT; (c) cryo-EM structure (PDB id: 5FN2) of the whole complex; (d) the same cryo-EM structure without NCT, Figure S3: Total number of clusters, five the most populated clusters (in orange), and the scores and energy contributions of the best scored poses, obtained for docking of exemplary conformation of one of substrate. Energetic contributions: el—electrostatic energy; vw—nonbonded interatomic pairwise interactions van der Waals energy; sf—surface energy term; ener—total energy being a sum of contributions with appropriate weights. All units are in kcal/mol. After pose clustering, the representative structures from five the most numerous clusters underwent refinement with ligand flexible side-chains, Figure S4: The scores and the binding energies obtained for the final conformations of both substrates, Aβ_43_ and Aβ_45_, after refinement with the ligand flexible side chains, Figure S5: The final poses of Aβ substrates docked to PS-1 and chosen for MD simulations. The pose numbers correspond to numbers in Figure S4. To explore maximal diversity of substrate poses, the maximally distinct poses among the lowest score poses were selected, with their N-termini being outside of the membrane and not interfering with other subunits of γ-secretase, Figure S6: The root-mean-square deviation (RMSD) and radius of gyration (R_g_) plots for exemplary MD simulations of Aβ in 10% (in blue) and 50% (in orange) cholesterol concentration. (a) RMSD plots for whole γ-secretase complex calculated for C_α_ atoms; (b) RMSD plots for transmembrane part of γ-secretase complex calculated for C_α_ atoms; (c) R_g_ plots for whole γ-secretase complex calculated for all atoms; (d) R_g_ plots for transmembrane part of γ-secretase complex calculated for all atoms, Figure S7: Root-mean-square fluctuations (RMSF) for particular subunits of γ-secretase and the substrate in 10% cholesterol membrane. RMSF values are averaged over four MD simulations for both substrates, Figure S8: Root-mean-square fluctuations (RMSF) for particular subunits of γ-secretase and the substrate in 50% cholesterol membrane. RMSF values are averaged over four MD simulations for both substrates, Figure S9: Root-mean-square fluctuations (RMSF) for particular subunits of γ-secretase and the Aβ_43_ substrate in membrane with 10% and 50% concentration of cholesterol. RMSF values are averaged over four MD simulations for both concentrations of cholesterol, Figure S10: Root-mean-square fluctuations (RMSF) for particular subunits of γ-secretase and the Aβ_45_ substrate in membrane with 10% and 50% concentration of cholesterol. RMSF values are averaged over four MD simulations for both concentrations of cholesterol, Figure S11: Number of hydrogen bonds between Aβ_43_ (without N-terminus) and PS-1 during 500 ns MD simulations conducted in the membrane with 10% and 50% of cholesterol, Figure S12: Number of hydrogen bonds between Aβ_45_ (without N-terminus) and PS-1 during 500 ns MD simulations conducted in the membrane with 10% and 50% of cholesterol.
