# Supplementary material for: The Role of Cholesterol in Amyloidogenic Substrate Binding to the γ-Secretase Complex
Source: Biomolecules. 2021 Jun 24;11(7):935. doi: 10.3390/biom11070935 (PMC8301813; doi:10.3390/biom11070935)
Supplement: Supplementary file 1 [file biomolecules-11-00935-s001.zip › biomolecules-1228195-supplementary.pdf]

Supplementary materials

The Role of Cholesterol in Amyloidogenic Substrate Binding to the γ-Secretase Complex

Urszula Orzeł, Jakub Jakowiecki, Krzysztof Młynarczyk *, Sławomir Filipek *

Faculty of Chemistry, Biological and Chemical Research Centre, University of Warsaw, 02-093 Warsaw, Poland.

* corresponding authors

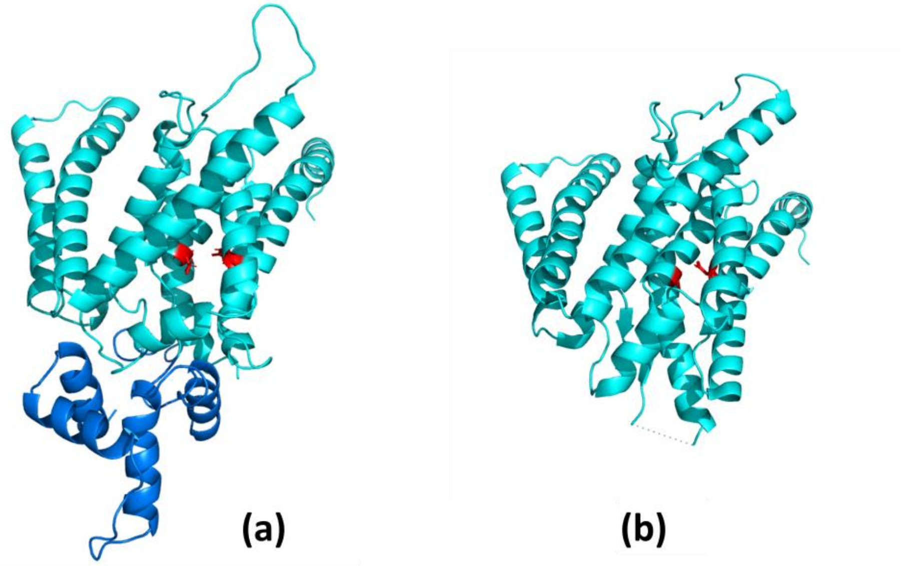

Figure S1. Comparison of two structures of PS-1. (**a**) The cryo-EM structure (Protein Data Bank PDB id: 5FN2) with reconstructed long cytosolic loop (residues 288-378, colored in blue) between helices TM6 and TM7 containing catalytic residues. (**b**) The cryo-EM structure (PDB id: 6IYC) with lacking residues 292-375 of cytosolic loop between helices TM6 and TM7. The catalytic residues are colored in red.

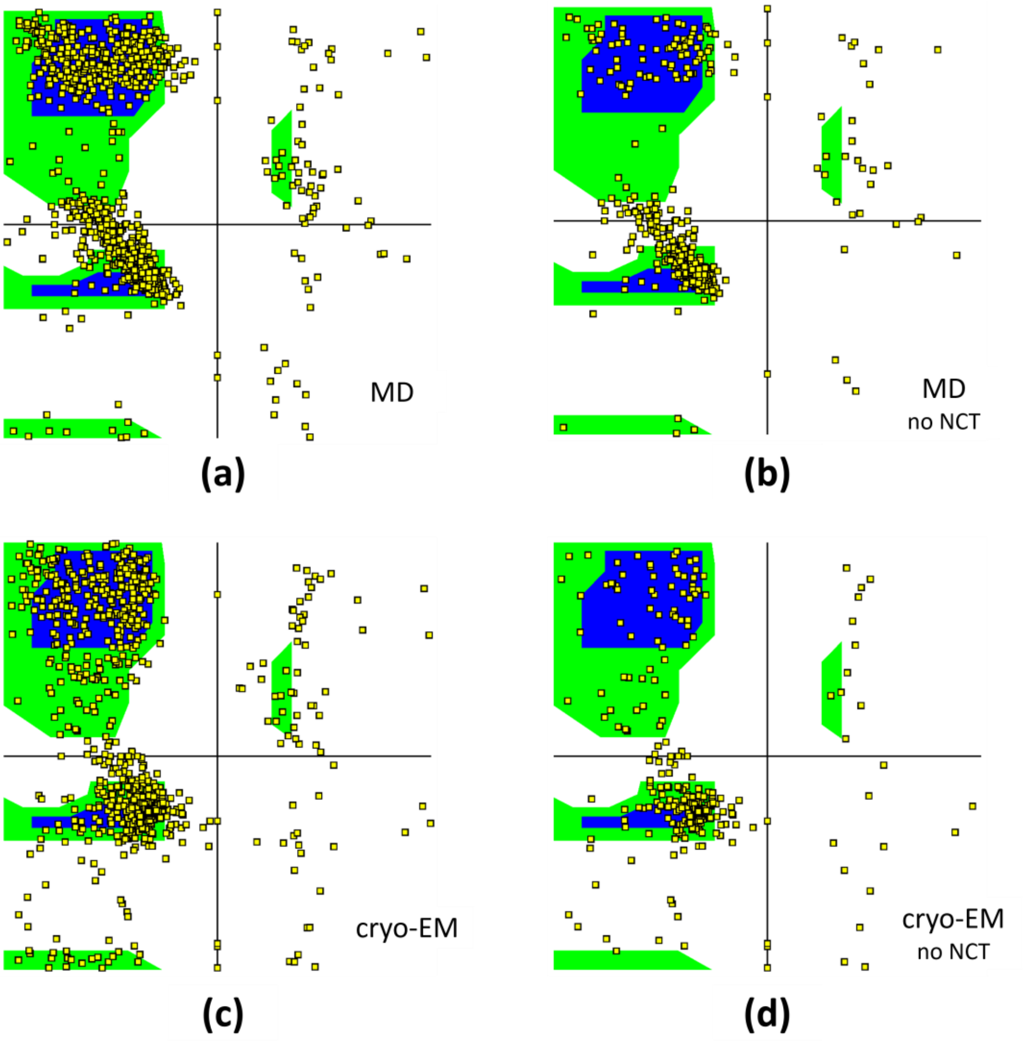

Figure S2. The Ramachandran plots for the structure of the γ-secretase complex without a substrate. (**a**) The structure after equilibration using 700 ns all-atom molecular dynamics (MD) simulation; (**b**) the same structure without NCT; (**c**) cryo-EM structure (PDB id: 5FN2) of the whole complex; (**d**) the same cryo-EM structure without NCT.

γ-secretase:Aβ43

Nr of clusters:	975				
cluster ID:	15	38	36	37	28
nr of conformations in cluster:	281	247	93	63	54

Score	el	vw	sf	ener	cluster ID
-38.7505	0.98168	-28.9523	-54.0667	-81.0557	36
-38.6791	0.169966	-17.2048	-61.6329	-78.4977	38
-38.2846	-1.47298	-28.651	-57.3204	-88.9173	15
-38.2616	-0.18131	-12.757	-66.5793	-79.6989	38
-36.7448	-4.54554	-35.0112	-47.7897	-91.892	15
-36.1077	-0.362963	-21.2908	-56.6992	-78.7159	38
-35.7485	-4.35595	-36.2386	-50.8155	-95.7661	15
-33.2242	-0.233914	-23.8579	-41.4548	-65.7805	37
-31.4510	-0.775991	-17.0677	-47.2543	-65.8739	37
-31.1352	-0.197986	-11.868	-52.7899	-65.0539	37

Figure S3. Total number of clusters, five the most populated clusters (in orange), and the scores and energy contributions of the best scored poses, obtained for docking of exemplary conformation of one of substrate. Energetic contributions: **el** - electrostatic energy; **vw** - nonbonded interatomic pairwise interactions van der Waals energy; **sf** - surface energy term; **ener** - total energy being a sum of contributions with appropriate weights. All units are in kcal/mol. After pose clustering, the representative structures from five the most numerous clusters underwent refinement with ligand flexible side-chains.

γ-secretase:Aβ43		
**Final pose**	**Score**	**Binding energy [kcal/mol]**
1	-194.75	-28.39
2	-208.57	-24.87
3	-177.86	-26.86
4	-306.53	-25.70
**average**	**-221.93**	**-26.46**

γ-secretase:Aβ45		
**Final pose**	**Score**	**Binding energy [kcal/mol]**
1	-147.92	-21.64
2	-170.32	-25.02
3	-132.88	-29.14
4	-174.67	-22.37
**average**	**-156.45**	**-24.54**

Figure S4. The scores and the binding energies obtained for the final conformations of both substrates, $A\beta_{43}$ and $A\beta_{45}$, after refinement with the ligand flexible side chains.

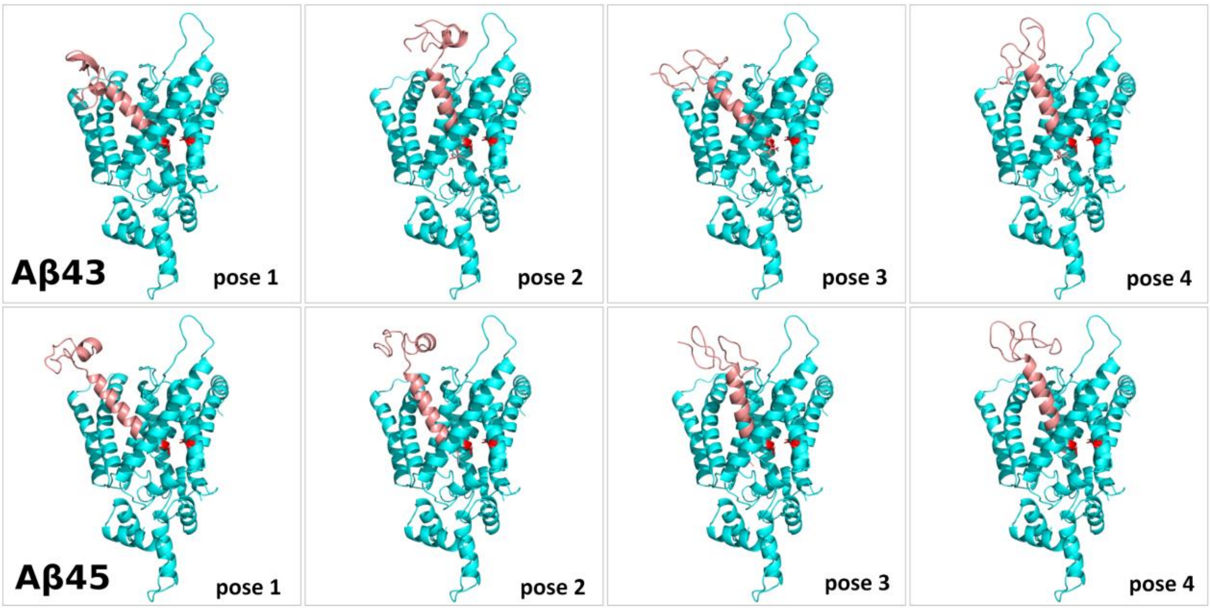

Figure S5. The final poses of Aβ substrates docked to PS-1 and chosen for MD simulations. The pose numbers correspond to numbers in Figure S4. To explore maximal diversity of substrate poses, the maximally distinct poses among the lowest score poses were selected, with their N-termini being outside of the membrane and not interfering with other subunits of γ-secretase.

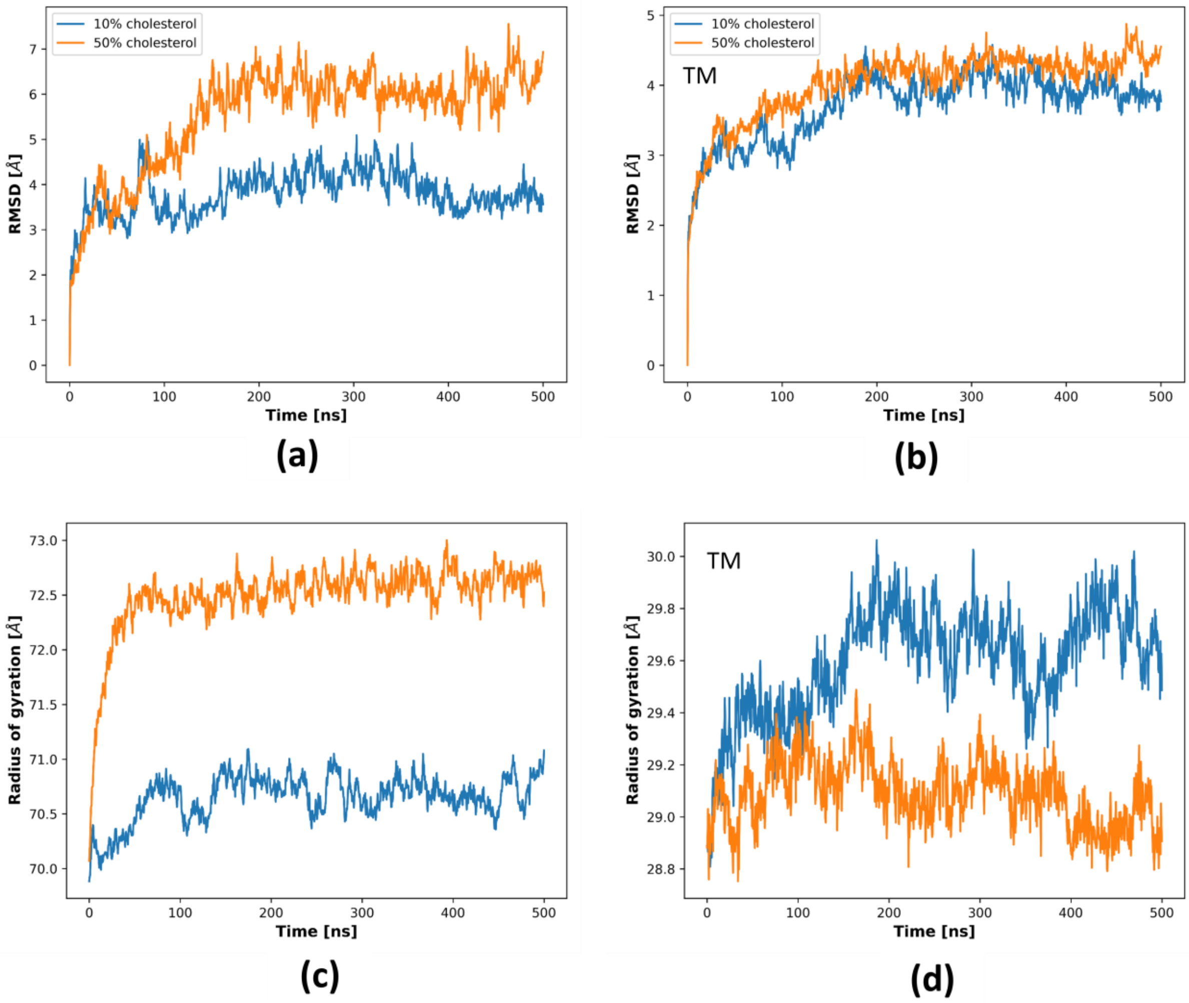

Figure S6. The root-mean-square deviation (RMSD) and radius of gyration (R_g) plots for exemplary MD simulations of Aβ in 10% (in blue) and 50% (in orange) cholesterol concentration. (**a**) RMSD plots for whole γ-secretase complex calculated for $C_α$ atoms; (**b**) RMSD plots for transmembrane part of γ-secretase complex calculated for $C_α$ atoms; (**c**) R_g plots for whole γ-secretase complex calculated for all atoms; (**d**) R_g plots for transmembrane part of γ-secretase complex calculated for all atoms.

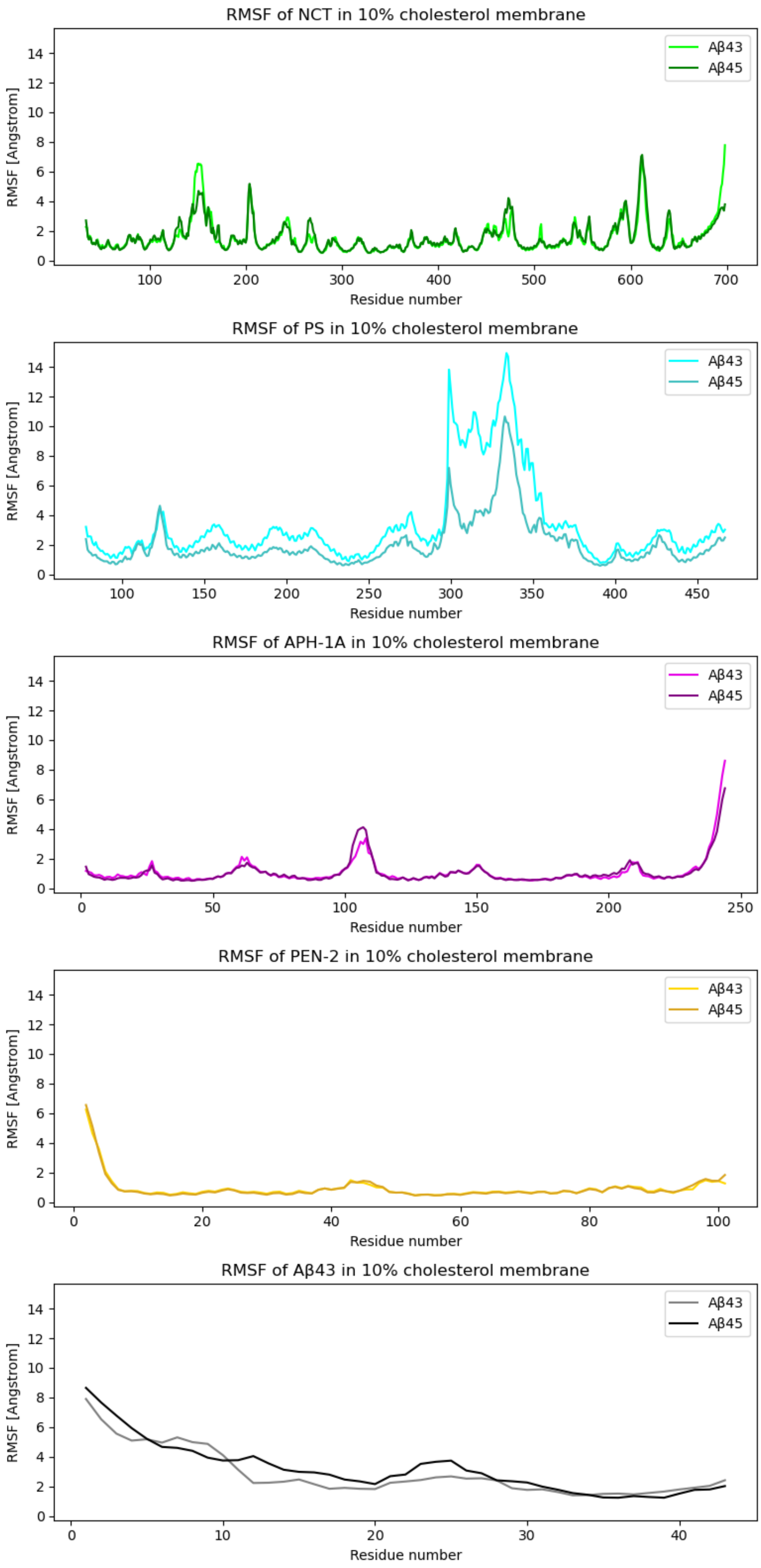

Figure S7. Root mean square fluctuations (RMSF) for particular subunits of γ-secretase and the substrate in 10% cholesterol membrane. RMSF values are averaged over four MD simulations for both substrates.

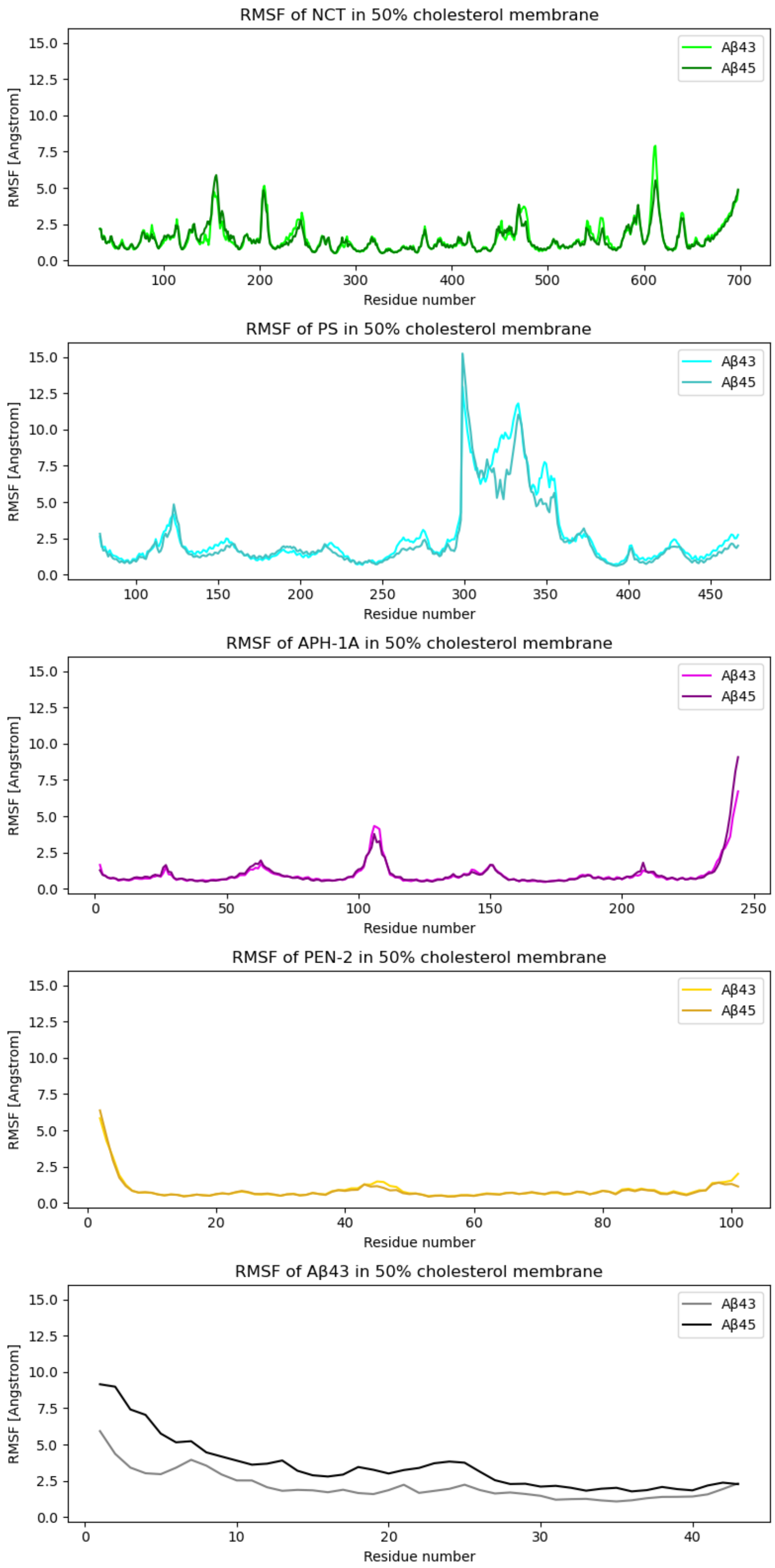

Figure S8. Root mean square fluctuations (RMSF) for particular subunits of γ-secretase and the substrate in 50% cholesterol membrane. RMSF values are averaged over four MD simulations for both substrates.

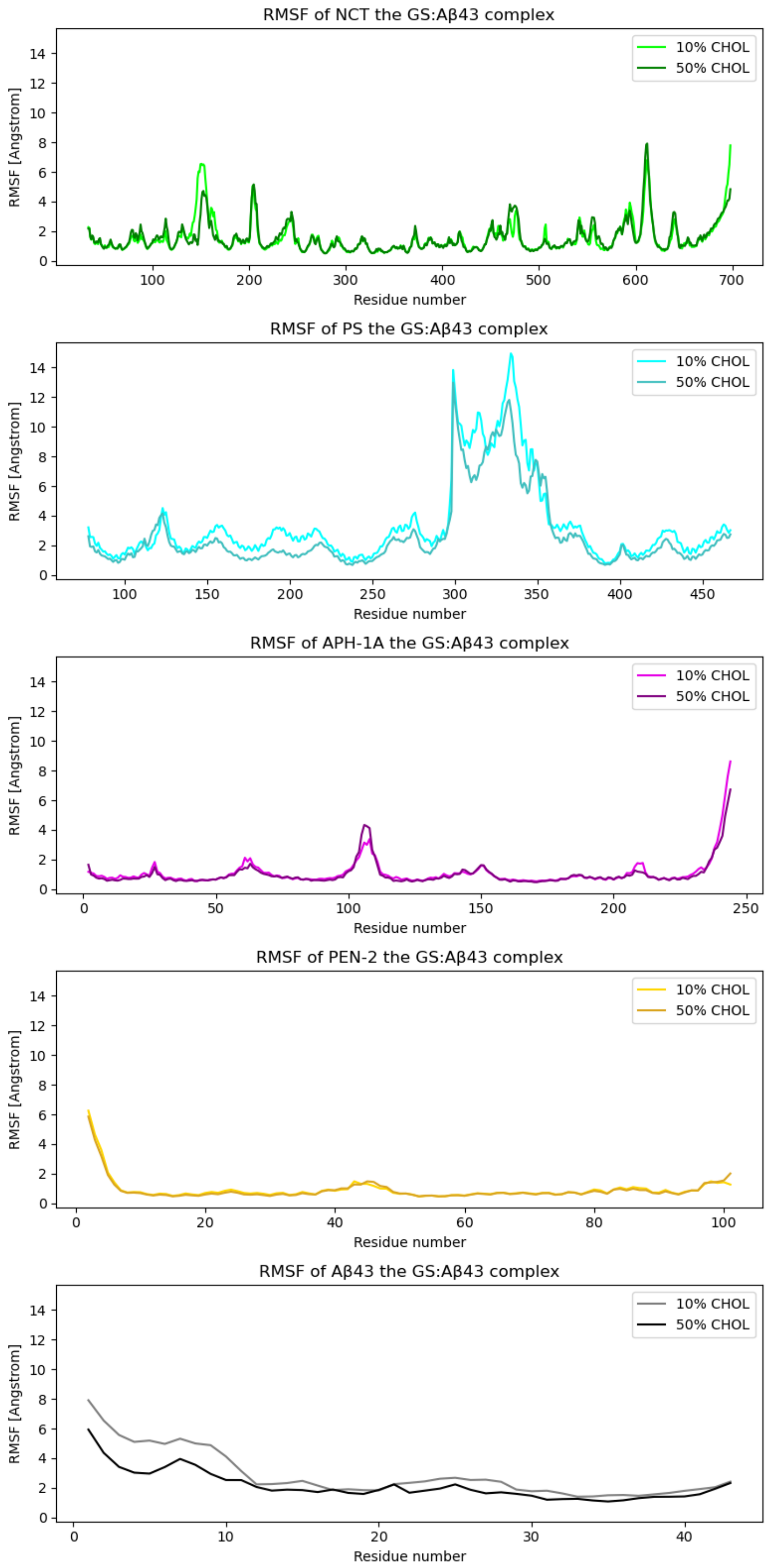

Figure S9. Root mean square fluctuations (RMSF) for particular subunits of γ-secretase and the $A\beta_{43}$ substrate in membrane with 10% and 50% concentration of cholesterol. RMSF values are averaged over four MD simulations for both concentrations of cholesterol.

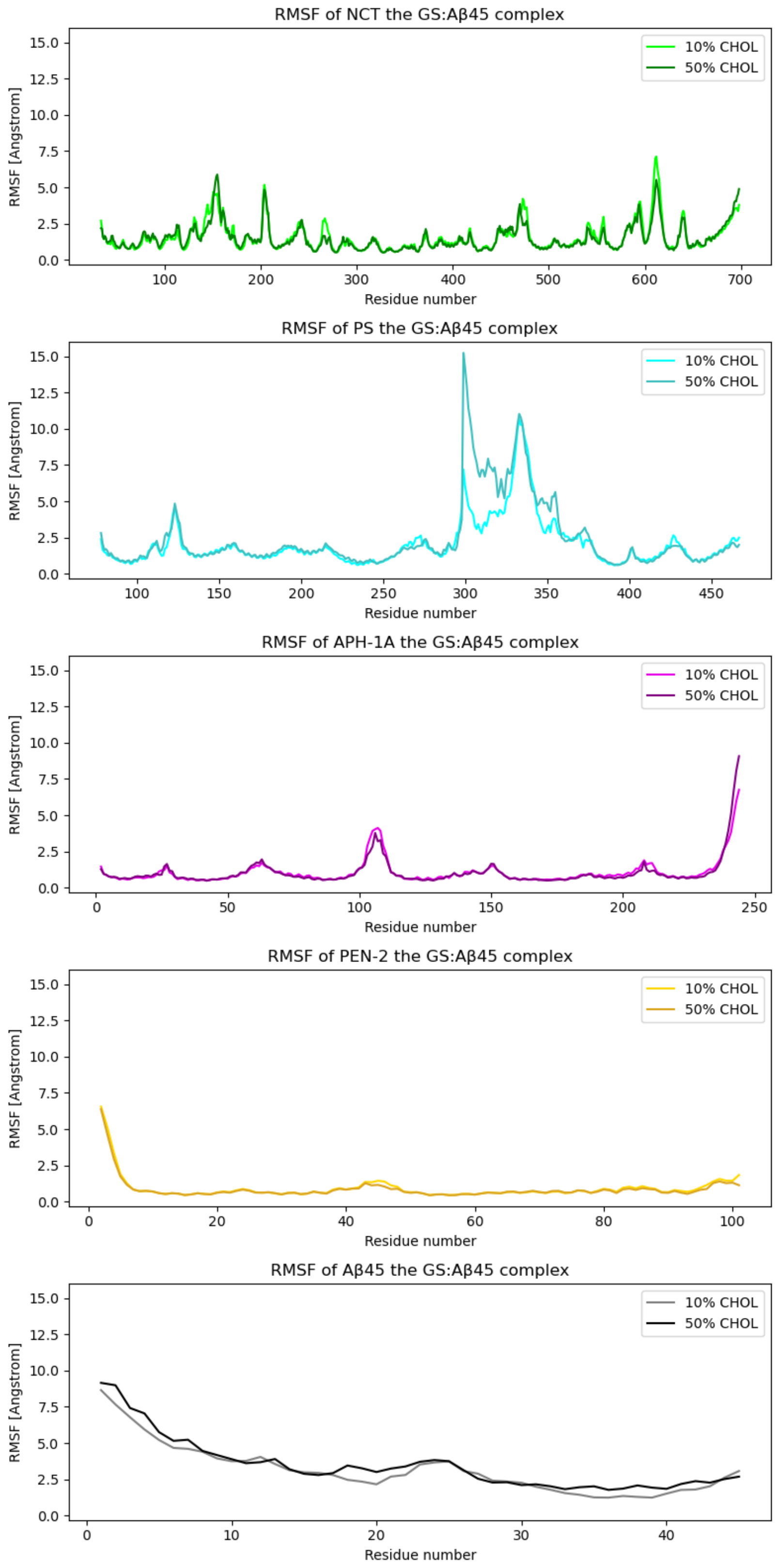

Figure S10. Root mean square fluctuations (RMSF) for particular subunits of γ-secretase and the $A\beta_{45}$ substrate in membrane with 10% and 50% concentration of cholesterol. RMSF values are averaged over four MD simulations for both concentrations of cholesterol.

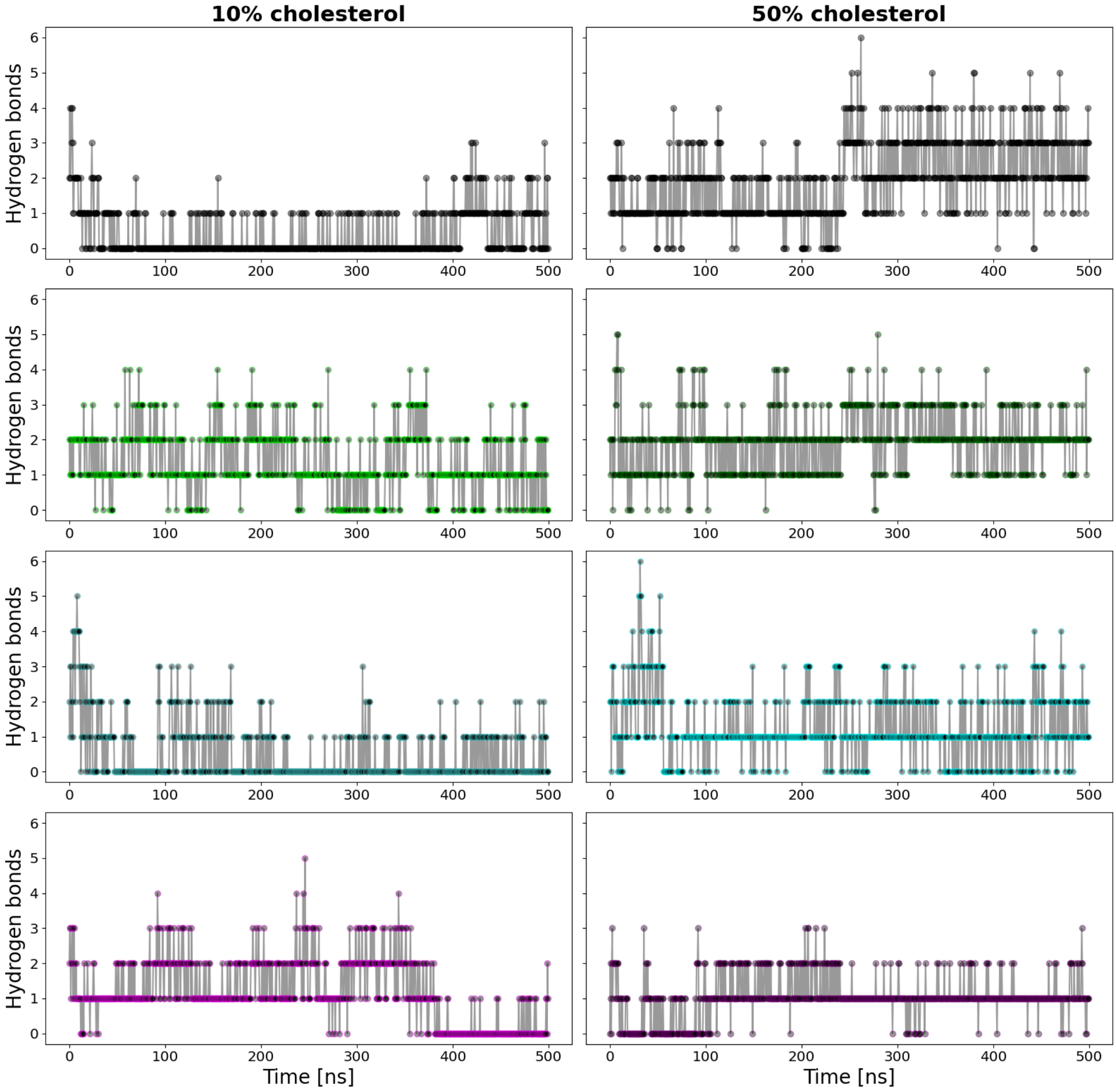

Figure S11. Number of hydrogen bonds between Aβ_{43} (without N-terminus) and PS-1 during 500 ns MD simulations conducted in the membrane with 10% and 50% of cholesterol.

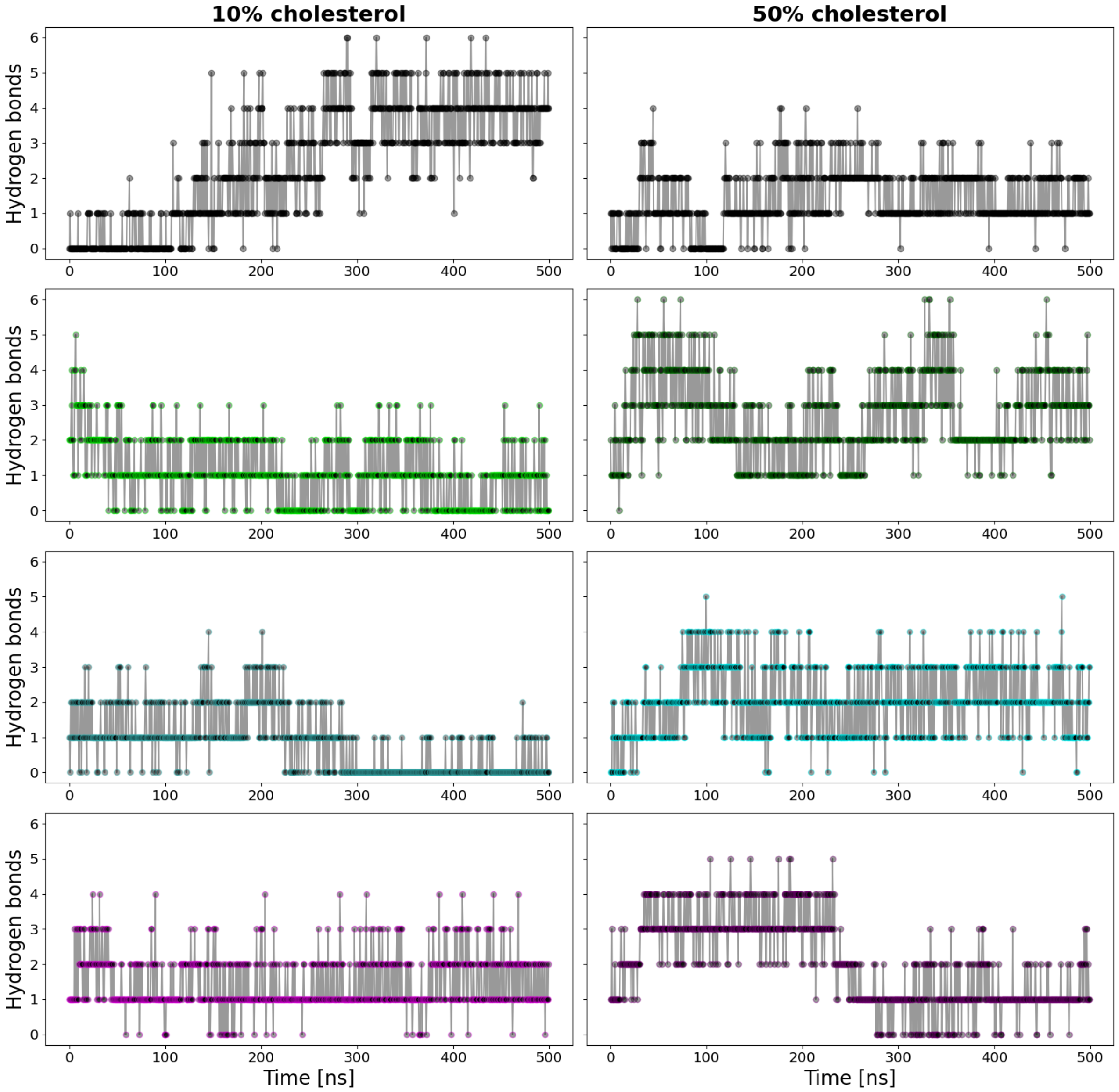

Figure S12. Number of hydrogen bonds between Aβ_{45} (without N-terminus) and PS-1 during 500 ns MD simulations conducted in the membrane with 10% and 50% of cholesterol.
